# 

**Published:** 1895-09-14

**Authors:** 


**Notices of Births, Deaths, and Marriages** are inserted in this column at a charge of 2s. 6d. for an announcement not exceeding four lines.

**Notice to Advertisers and Subscribers.**

All Advertisements, Orders for Copies of The Hospital and Business Communications should be addressed to **The Manager, NOT TO THE EDITOR,** The Hospital, 428, Strand, London, W.C.

The Cost of Subscription, post free, is as follows:—Per week, 2½d.; per quarter, 2s. 8d.; per half-year, 5s. 3d.; per annum, 10s. 6d. Foreign:—Per Annum, 15s. 2d.; payable, in all cases, in advance.

### NOTICE TO CORRESPONDENTS.

All MS., Letters, Books for Review, and other matters intended for the Editor should be addressed The Editor, The Lodge, Porchester Square, London, W.

The Editor cannot undertake to return rejected MS., even when accompanied by stamped directed envelope.

**"Burdett's Hospital Annual," 1895,**

Sixth year of publication. 950 pp. Scarlet cloth, gilt lettered. Price 5s. A most complete guide to British, Colonial, Indian, and American Hospitals, Dispensaries, Nursing and Convalescent Institutions, and Asylums. A few copies of the "Annual" for 1894 may still be obtained on application to the Publishers, The Scientific Press (Limited), 428, Strand, London, W.C.

**NOTICE.—Vol. XVII. of "The Hospital" (October, 1894, to April, 1895), in handsome cloth binding, is on sale at the Office of this Journal. Price 6s. Cases for binding the Numbers contained in this Volume, 1s. 6d. Index to Vol. XVII., 2½d., post free.**

**The Monthly Part for September is now ready, price 6d.; by post, 8d.**

### SCRAPS AND GLEANINGS.

No less than 3,684 shillings have been collected in the 1s. subscription list towards the funds of the Hertford Infirmary.

The Employés' Committee of the Bradford Joint Hospital Fund gave their fourth annual gala on Saturday in Lister Park.

At the Littlehampton Annual Hospital Sunday Amalgamated Demonstration, which has just taken place, no fewer than 2,539 coppers were collected.

The Liquor Carnis Company (28, Farringdon Street, E.C.) are offering a very handy pocket foot rule to be sent, free of cost, to anyone applying by postcard for one to the firm.

Pending the erection of extended premises, the Hertford and Ware Joint Hospital Board have decided to renew the lease of the temporary hospital at Little Gobions for another year.

There is much difficulty being experienced by the Exmouth District Council regarding a site for the Isolation Hospital about to be erected for that locality. Serious objections are being raised to the site suggested.

The National Temperance Congress, which will be held at Chester the end of this month, has been convened by the National Temperance League, and will be presided over by Sir Benjamin Ward Richardson, M.D., F.R.S.

The Lord Lieutenant of Devonshire has consented to convene a public meeting for the purpose of obtaining further contributions to the extension fund of the Devon and Exeter Hospital. About £4,000 has so far been collected for this purpose.

The question of the erection of a temporary hospital at Leith has just come, as an adjourned petition by the Edinburgh Corporation, before the authorities at Leith. An animated discussion ensued, and serious local objections were raised to this scheme being carried out.

The result of the Hospital Saturday collection at Lewes proved highly satisfactory to all concerned. The Mayor of the town not only took upon himself a great deal of trouble personally in the scheme, but he also generously took upon himself some of the financial responsibility too.

The annual meeting of governors and subscribers of the Alexandra Hospital, Woodhall Spa, has just been held. Outstanding debts of £60 still remain on the furnishing of the Clarence Ward. Whilst the annual subscription list has somewhat increased, donations have slightly fallen off.

Mr. Chaplin, President of the Local Government Board, has paid an official visit to the Farm Colony of the Salvation Army at Hadleigh, Essex. The President, who was accompanied by Sir Hugh Owen, Secretary to the Board, spent several hours examining the various departments of the Farm.

At the Worthing Infirmary the year commenced with a balance due to the treasurer of £225, and a liability of £62, the latter being caused by the necessity of laying new water-pipes throughout the building, but these liabilities have been discharged, Mr. Henry Smart having generously given the sum of £300 for that purpose.

A fete planned on an extensive scale was held recently upon the glebe land, Willesden Green, in aid of the Willesden Cottage Hospital and the St. Monica's Hospital for Sick and Crippled Children at Brondesbury. Among other attractions here were a bakers' exhibition, a flower show, a horse show, and military tournament.

The sixty-ninth annual meeting of the subscribers to the North Devon Hospital has just been held. Legacies to the extent of £2,000 have been bequeathed to the institution during the period under review. The house surgeon's report shows that 697 in-patients and 2,857 out-patients have been admitted to the benefits of the institution during the year.

A literary offshoot from *English Sports* has made its appearance in the magazine world. This is entitled *Swimming*, and is the only journal devoted to the subject. The organizers are hereby endeavouring to popularize and raise the tone of swimming as a sport and pastime. An insurance scheme in connection with the venture is a new departure.

The attention called to "Filthy Railway Carriages" by a correspondent of *The British Medical Journal* should be productive of some reform in a matter which is of universal interest. The disgusting condition of the floors of carriages on the metropolitan lines is a perpetual trial to all cleanly men and women obliged by circumstances to travel underground.

The annual report of the Morley House Convalescent Home, at St. Margaret's Bay, Dover, shows that 798 patients have been received during the year, giving an increase of 132 over the previous one. The home is free from debt, but the number of patients seeking admission is growing so rapidly that some steps will have to be taken in the near future to increase the accommodation.

A bazaar was opened quite recently on the Esplanade, Rothesay, in aid of the proposed new non-infectious hospital. The bazaar was opened for four days for this purpose. Good and useful work has for some time past been done at this hospital, but the accommodation was found to be of such a limited character that the committee felt called upon to provide larger and more suitable premises.

The proprietors of Mellin's Food for Infants and Invalids have just issued a pretty little painting book. Not only is this work useful as a painting lesson for students, but it has the further object of giving purchasers of coloured pictures, printed in England, an idea of the manner of their reproduction. The book is obtainable of Mather and Crowther, 12, Bridge Street, Ludgate Circus.

Tenders for the erection of the North-Eastern Counties Convalescent Home have been at a much higher rate than was at first anticipated. The position, however, has been fully explained, and one of the trustees is urging that each centre should endeavour to raise funds by means of bazaars, &c. The Manchester Unity of Oddfellows have offered the committee the sum of £1,300 towards the cost of the new erection at the rate of 3 per cent. interest.

Messrs. Carlisle and Co. prove themselves advocates of temperance in a very practical manner. Considering that the majority of accidents on their steamers were due to want of sobriety, they stipulated some years ago that the captains should become total abstainers, offering an extra bonus of £100 to those who kept their ships free from accident, and £50 to those on whose vessels not more than £25 was spent on repairs due to accident.

### The Student of Medicine.

### APPOINTMENTS.

C. M. Fegen, M.R.C.S., L.R.C.P., and Diploma in State Medicine, appointed Medical Officer of Health to the Ampthill Rural District Council. Dr. Halligan, appointed Medical Officer for the Ballyroan Dispensary District of the Abbeyleix Union. H. H. Haward, B.A.Cantab., L.R.C.P., M.R.C.S., appointed Clinical Assistant in the Ear Department of St. Thomas's Hospital. A. L. Home, L.R.C.P., M.R.C.S., appointed House Surgeon to St. Thomas's Hospital. L. L. Jenner, M.A., M.B., B.Ch.Oxon., M.R.C.P. (extension), appointed Resident House Physician to St. Thomas's Hospital. F. G. Layton, L.R.C.P., M.R.C.S., appointed non-resident House Physician to St. Thomas's Hospital. M. M. Louden, M.D., appointed Public Vaccination Officer for the Arundel District of the East Preston Union. H. Macintyre, M.B., C.M.Glasg., appointed Assistant Medical Officer to Shoreditch Infirmary. E. Orchard, M.B., C.M.Aberd., appointed Medical Officer of Health to the Kingussie and Irish Parish Council. E. W. Palin, M.A., M.B., B.Ch.Oxon, L.R.C.P., M.R.C.S., appointed Clinical Assistant in the Ear Department of St. Thomas's Hospital. J. L. Prain, L.R.C.P., M.R.C.S., appointed Assistant House Surgeon to St. Thomas's Hospital. P. Macleod Yearsley, F.R.C.S., appointed Clinical Assistant to the Central London Throat and Ear Hospital.

### VACANCIES.

The following vacancies are announced this week, the amount of salary in each case being given after the name of the institution:—

### Resident Medical Officers.

Chesterfield and North Derbyshire Hospital and Dispensary, Chesterfield.—Resident House Surgeon; tenable for two years. Salary £100 per annum, with board, apartments, and laundries. Applications and testimonials to the Secretary before September 19th.

City of London Hospital for Diseases of the Chest, Victoria Park, E.—House Physician. Board and residence and allowance for washing provided. Appointment for six months. Applications to the Secretary by September 12th.

Great Yarmouth Hospital.—House Surgeon; must be doubly qualified and able when required to give lectures for probationer nurses. Salary £90 per annum, with board and lodging. Applications and testimonials to R. F. E. Ferrier, Honorary Secretary, before September 14th.

Rotherham Hospital and Dispensary.—Assistant House Surgeon; doubly qualified, and registered. No salary, board, lodging, and washing. Applications and testimonials to the House Surgeon by October 1st.

### Non-Resident Medical Officers.

City of London Hospital for Diseases of the Chest, Victoria Park, E.—Assistant Physician; must be M. or F.R.C.P.Lond. Applications to the Secretary by September 14th.

Glasgow Maternity Hospital.—Obstetric Physician and Assistant Obstetric Physician. Applications to Arthur Forbes, Secretary, 146, Buchanan Street, Glasgow, by November 8th.

St. Bartholomew's Hospital and College.—Assistant Demonstrator of Chemistry. Applications to Thomas W. Shore, Warden, before September 21st.

### UNIVERSITIES AND COLLEGES.

### Examining Board in England by the Royal Colleges of Physicians and Surgeons.

The following gentlemen passed the First Examination of the Board in the subjects indicated under the "Four Years'" Regulations:—

Passed in Part I. Chemistry, including Chemical Physics.—V. S. A. Bell, University of Cambridge and St. Bartholomew's Hospital; P. M. S. Bennett, Middlesex Hospital; C. H. D. Balteel, King's College, London; T. L. Butler, St. Mary's Hospital; E. C. Dean, University College, London; F. A. Hart, Middlesex Hospital; T. A. King, University of Cambridge; L. A. Lewis, University College, Cardiff; E. A. Nicholls, London Hospital; W. Nicholls, St Bartholomew's Hospital; C. A. Scott, Charing Cross Hospital; E. R. L. Thomas, London Hospital; W. W. Williams, University College, London.

Passed in Part II. Materia Medica.—A. J. Andrew, St. Bartholomew's Hospital; A. J. Bennetts, St. Mary's Hospital; G. T. Brundrett, Owens College, Manchester; P. H. Collingwood, St. Thomas's Hospital; W. J. E. Davies, St. Thomas's Hospital; E. C. Dean, University College, London; H. G. C. Dring, St. Bartholo-

**THE STUDENT OF MEDICINE**—*continued.*

mew's Hospital; E. P. Du Heaume, St. Bartholomew's Hospital; R. T. Ellis, Yorkshire College, Leeds; J. S. Gaymer, St Bartholomew's Hospital; H. A. Good, King's College, London; P. O. Grüber, St. Bartholomew's Hospital; J. R. Hatfield, Yorkshire College, Leeds; W. L. Hay, King's College, London; A. J. Hayes, Middlesex Hospital; T. E. Holman, Guy's Hospital; J. Howells, London Hospital; C. W. Iliffe, Owens College, Manchester; A. Jones, London Hospital; N. P. King, Charing Cross Hospital; A. J. Knowlton, Middlesex Hospital; F. S Langworthy, St. George's Hospital; G. E Leary, Mason College, Birmingham; E. T. Longhurst, St. Mary's Hospital; C. H. Maskew, Mason College, Birmingham; W. G. Mitchell, Guy's Hospital; W. Nicholls, St. Bartholomew's Hospital; F. H. Nimmo, St. Bartholomew's Hospital; E. H. Pomeroy, Guy's Hospital; H. L. Porteous, Middlesex Hospital; C. G. Pugh, Middlesex Hospital; E. A. Quicke, Mason College, Birmingham; S. T. G. Ransford, St. Mary's Hospital; J. A. A. Rouillard, St. Thomas's Hospital; C. A. Scott, Charing Cross Hospital; A. L. H. Smith University College, London; A. P. Square, Middlesex Hospital; P. H. Stratton, Mason College, Birmingham; G. L. Thornton, St. George's Hospital; A. S. White, Ledwich School, Dublin; J. M. Wood, Middlesex Hospital; C. P. Woodstock, Anderson's College, Glasgow; A. Woollcombe, St. Bartholomew's Hospital.

Passed in Part III. Elementary Anatomy and Elementary Physiology.—G. T. Brundett, Owens College, Manchester; C. H. D. Bulteel, King's College, London; E. C. Dean, University College, London; [illegible]. M. Dowling, Catholic University of Ireland, Dublin; J. Evans, St. George's Hospital; A. Hawkins, University of Cambridge and St Bartholomew's Hospital; S. Hey, University of Cambridge and St. Bartholomew's Hospital; T. A. King, University of Cambridge and St. Thomas's Hospital; W. Marsden, Yorkshire College, Leeds; H. H. Mayor, Owens College, Manchester; J. H. Mort, University of Cambridge; G. B. Nicholson, University of Cambridge; C. H. Sanders, St. George's Hospital; F. A Simpson, University of Cambridge and St. Mary's Hospital; G. C. Walker University College, Liverpool.

Passed in Physiology Only.—T. McCarthy Queen's College, Cork.

The following gentlemen, having passed the necessary examinations, have been admitted by the two Royal Colleges Diplomates in Public Health: S. G. Allen, Surgeon-Captain Army, M R.C.S.Eng., L R C P.-Edin., St. Mary's Hospital; C. Childs, B.A., M D Oxon., L.R.C P.-Lond., M.R.C.S.Eng., St. George's Hospital and Oxford and Bonn Universities; F. Chown, M.B Lond. L.S.A.Lond., St Mary's Hospital; H. S. Fremlin, L.R C.P Lond., M.R C.S.Eng., Westminster Hospital; M. Gepp, L.R.C.P. and S.Edin., L F.P. and S.Glasg. St. Bartholomew's Hospital; E. O. Hopwood, M.A., M D.Oxon., University College, Owens College and Royal Infirmary, Manchester; D. O. Kerr, M.B., C.M.-Edin., Edinburgh University; E T. Larkham, L R.C.P.Lond., M.R.C.S.Eng., Middlesex Hospital; A. Lindow, M.R.C.S.Eng., L S.A.-Lond., King's College; W. P. Norris, M.B.Melb., Melbourne University; W. W. Pryn, Surgeon Royal Navy, M.R.C.S.Eng., L.S A.Lond., Guy's Hospital; W. W Shrubsall, L R.C.P. and S Edin., L.F.P. and S.-Glasg., Edinburgh and King's College; H. Vallance, M.R.C.S.Eng., London and Guy's Hospitals; A. W. Wigmore, L.R.C P Lond., St. Mary's Hospital; A. W. Williams, M B., C.M Edin., Edinburgh University, London, King's College, and St. Thomas's Hospitals. Eleven gentlemen were referred for six months.

